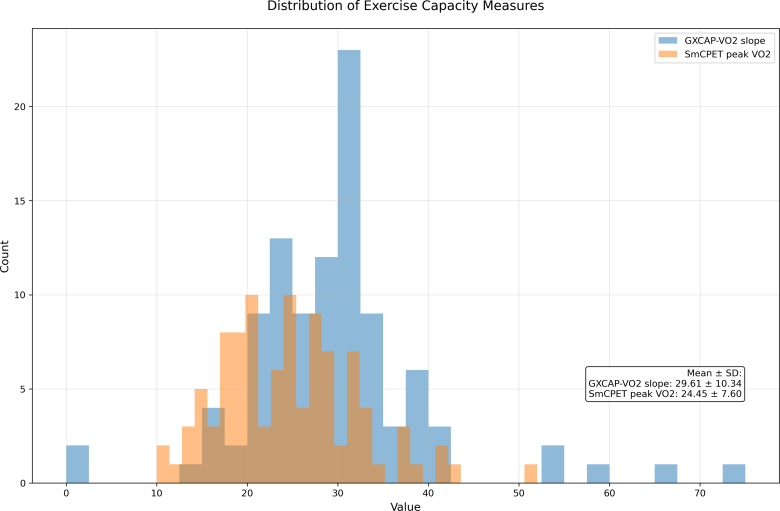# Preoperative submaximal cardiopulmonary exercise testing and its association with early postoperative complications

**DOI:** 10.1016/j.bjao.2025.100407

**Published:** 2025-04-24

**Authors:** Zyad J. Carr, Jean Charchaflieh, Andres Brenes-Bastos, Hugo He, Hung-Mo Lin, Amanda Jankelovits, Emily Gu, Jill Zafar, Fed Ghali, Wei S. Tan, Paul Heerdt

**Affiliations:** 1 Yale University, School of Medicine, New Haven, CT, USA; 2 Department of Anesthesiology, Yale New Haven Hospital, New Haven, CT, USA; 3 Yale Center for Analytical Sciences, New Haven, CT, USA; 4 Department of Anesthesiology, Bridgeport Hospital, Bridgeport, CT, USA; 5 Department of Surgery, Yale New Haven Hospital, New Haven, CT, USA

**Keywords:** early postoperative complications, preoperative evaluation, preoperative functional capacity, preoperative risk stratification, submaximal cardiopulmonary exercise testing

## Abstract

**Background:**

Early postoperative complication risk prediction would enhance perioperative surveillance and resource allocation. Reports have described brief submaximal cardiopulmonary exercise testing (CPET) for the routine assessment of cardiopulmonary disease. Compared with conventional CPET, it can be performed in 6 min and is used to predict peak CPET measurements. We aimed to determine whether submaximal CPET-derived measures outperform structured surveys in early postoperative complication detection.

**Methods:**

An institutional review board-approved, single-centre, open-label, clinical device trial was conducted. A total of 101 participants undergoing noncardiac surgery, aged >60 yr, with revised cardiac risk index ≤2, self-reported metabolic equivalents >4 (METs in ml O_2_ kg^−1^ min^−1^; self-endorsed reliably climbing two flights of stairs), were enrolled. Participants completed a subjective METs assessment, Duke Activity Status Index, and submaximal CPET that derived peak oxygen uptake (VO_2_, ml O_2_kg^−1^ min^−1^), METs, and gas exchange-derived pulmonary capacitance (GXCAP, in ml O_2_ beat^-1^ kPa^-1^). Elastic net regularisation machine learning identified feature importance among study measures for the primary endpoint (Postoperative Morbidity Survey [POMS] ≥1), secondary endpoints (cardiac, pulmonary and renal domains of the POMS [POMS-CPR ≥1]), and length of stay. Adjusted multivariable regression models were used to identify significance.

**Results:**

Of 101 participants, 53 (52.4%) had POMS ≥1. GXCAP to peak VO_2_ slope (GXCAP-VO_2_) was associated with POMS ≥1 (OR_adj_ 0.94; 95% CI 0.89–0.99; *P*=0.011) and increasing length of stay (OR_adj_ 0.98; 95% CI 0.96–0.99; *P*=0.01). GXCAP-VO_2_ slope (OR_adj_ 0.93; 95% CI 0.88–0.99; *P*=0.015) was associated with POMS-CPR ≥1.

**Conclusions:**

Compared with structured surveys (subjective METs or Duke Activity Status Index) or conventional peak CPET values (VO_2_ or METs), a novel measure, GXCAP-VO_2_ slope, offered superior early postoperative complication discrimination in low-morbidity subjects. These preliminary findings support GXCAP-VO_2_ slope as a compelling investigational target for early postoperative complication risk, supporting the use of CPET to enhance early postoperative complication prediction.

**Clinical trial registration:**

NCT05743673.

With an incidence as high as 75.1% after major elective surgery, early postoperative complications (EPCs) impact healthcare costs and length of stay.^1^^,^^2^ Preoperative EPC risk prediction would facilitate patient identification for enhanced perioperative surveillance, intervention development, and strategic resource allocation. The usual approach for preliminary preoperative risk assessment is provider-driven subjective assessment of functional capacity, frequently measured as multiples of metabolic equivalents (METs, where 1 MET=3.5 ml O_2_ kg^−1^ min^−1^). A reported functional capacity of less than two flight of stairs (<4.6 METs) has been associated with higher risk of cardiac death and adverse perioperative events.^3^, ^4^, ^5^, ^6^ However, provider-driven assessment has demonstrated poor sensitivity for METs ≤4 and has not improved perioperative outcomes.^7^^,^^8^ Thus, there is a need for new methods to both precisely characterise functional capacity, enhance preoperative EPC detection, and predict major perioperative cardiovascular complications.^8^

Cardiopulmonary exercise testing (CPET) has been acknowledged as a cost-effective and essential measure of cardiopulmonary assessment by the American Heart Association.^9^^,^^10^ CPET measures predict major perioperative complications including length of stay, intensive care admission, and perioperative complications.^11^, ^12^, ^13^, ^14^, ^15^ Furthermore, CPET measures, such as the anaerobic threshold, have shown efficacy in EPC detection, as measured by the Postoperative Morbidity Survey (POMS).^13^ In published reports, CPET peak oxygen uptake (peak VO_2_, in ml O_2_kg^−1^ min^−1^) identified moderate to severe postoperative complications but not major postoperative cardiovascular outcomes.^7^ In the USA, CPET has not been widely adopted in preoperative screening, possibly owing to limited access, technical training barriers, and time constraints.^16^ Structured surveys, such as the Duke Activity Status Index (DASI), have emerged as a CPET alternative for the preoperative characterisation of functional capacity and cardiovascular risk.^17^ Although these surveys have shown efficacy in predicting high or low risk for postoperative cardiovascular complications, studies have documented overestimation bias or primarily focused on very high-risk populations.^18^, ^19^, ^20^

As an alternative to preoperative CPET or structured surveys, recent reports have described the diagnostic utility of brief submaximal cardiopulmonary exercise testing (smCPET), applied to the routine assessment of patients with cardiopulmonary disease.^21^, ^22^, ^23^ Brief smCPET utilises brief time-limited graded exercise (3 min), and peak value extrapolation using the oxygen uptake efficiency slope. Brief smCPET has been validated to CPET, excels at identifying cardiopulmonary impairment and discriminating congestive heart failure and pulmonary hypertension disease phenotypes,^24^, ^25^, ^26^ conditions which are associated with significant perioperative morbidity. One notable smCPET measure is gas exchange-derived pulmonary capacitance (GXCAP). GXCAP is calculated as O_2_ pulse × *P*_ET_co_2_, where O_2_ pulse = VO_2_ (in ml O_2_min^−1^)/heart rate (beats min^−1^) and *P*_ET_co_2_ is the partial pressure of exhaled carbon dioxide (in kPa). GXCAP is calculated continuously on a beat-by-beat basis during CPET, with key measurements including its peak exercise value, nadir value, and slope characteristics during exercise challenge and recovery. GXCAP correlates with invasive measurement of pulmonary capacitance (r=0.86), a prognostic factor for pulmonary hypertension mortality, and smCPET outperforms echocardiography in the discrimination of pulmonary hypertension phenotypes.^21^ If smCPET-derived measures can accurately identify participants with higher EPC risk, this would provide a foundation for developing targeted interventional research aimed at reducing these events.

The primary objective was to compare the effectiveness of two preoperative methods for the detection of EPCs: a usual approach using structured surveys (subjective METs, DASI) *vs* brief smCPET measures. The primary endpoint was EPCs, as measured by postoperative day 1, 3, and 5 POMS aggregated score. Secondary endpoints included isolated cardiac, pulmonary, and renal domains of the POMS (POMS-CPR) and length of stay. We hypothesised that smCPET measures would more effectively identify EPC risk, when compared with structured surveys.

## Methods

### Study design and setting

This prospective open-label clinical device investigation was approved by the Yale University Institutional Review Board (IRB#2000033885; ClinicalTrials.gov Registry NCT05743673; Principal Investigator: ZJC; date of registration: 12 May 2023). The trial was registered before participant enrolment. Recruitment was conducted from June 2023 to June 2024.

### Study population

Participants who met the following inclusion criteria were enrolled: age >60 yr, revised cardiac risk index of ≤2, and self-endorsed subjective METs of >4 (defined as the ability to reliably climb two flights of stairs), presenting for noncardiac surgery with general anaesthesia.^27^ Given the novelty of smCPET in preoperative evaluation, revised cardiac risk index score ≤2 was mandated by the institutional review board for patient safety purposes. Exclusion criteria were adapted to maintain current standard of care practices in preoperative evaluation. Participants were excluded if there was recorded severe or critical valvular heart disease, active exertional angina, non-ambulation, gait abnormalities, end-stage renal disease, severe peripheral vascular disease, or neurological motor deficits. Additionally, non-English speaking participants, those under legal guardianship, and participants documented to lack personal healthcare decision-making capacity were excluded.

### Sample size calculation

An *a priori* power analysis was conducted to determine the minimum sample size for detecting the relationship between submaximal cardiopulmonary exercise measures and EPCs. Based on the previous literature, the expected EPC incidence, as measured by the POMS, was estimated at 40% in this relatively healthy cohort (revised cardiac risk index score ≤2).^2^ Using G∗Power analysis with an effect size of 0.4 (medium by Cohen's standards), alpha 0.05, and power 0.80, the minimum required sample size was estimated to be 52 participants. To account for the inclusion of up to three significant covariates in the adjusted model, the sample size was increased by 10% per covariate, resulting in final minimum sample size of 68 participants. Accounting for a 10% drop out rate, a minimum number of 75 participants was selected.

### Submaximal cardiopulmonary exercise testing protocol

#### Equipment

The FDA-approved Shape II® system (Shape Medical Systems, Inc., White Bear Lake, Minnesota, USA) is a compact cardiopulmonary breath-by-breath exercise testing system that uses submaximal exercise effort (3 min) to generate multiple quantitative measures of actual (attained) and predicted peak cardiopulmonary performance. Using previously published calculations utilising the oxygen utilisation efficiency curve, peak exercise values are automatically predicted by submaximal exercise effort. The calculations and the device have been previously validated to CPET measurements.^28^^,^^29^ The compact design of the device allows all the necessary equipment to be placed on a standard rolling cart and it was deployed in a preoperative clinic examination room (2.4 × 2.4 m).^30^ The device provides an option for either time- or symptom-limited assessment. The timed session was selected for all study participants and requires a total of 6 min: 2 min of baseline resting data, 3 min of escalating exercise using a stationary stair-step, and 1 min of seated or standing recovery data (see Supplementary Table S1 for detailed description of smCPET performance and measurements). All structured survey instruments were adapted from prior publications.^31^, ^32^, ^33^

#### Study procedure

After pre-screening and within 6 weeks of surgical procedure, eligible participants were contacted by phone and invited for in-person written informed consent, preoperative evaluation, and experimental session. Session time included: (1) a seven-question subjective METs assessment; (2) 12-question DASI survey; and (3) timed smCPET (6 min). Upon arrival, participants' height, weight, and vital signs (heart rate, blood pressure, and pulse oximetry) were measured, followed by informed written consent and smCPET instruction (∼5 min). Structured surveys and smCPET were sequentially performed, followed by a preoperative evaluation. Participants were monitored for recovery in the seated position for 5 min after the termination of smCPET. Participants were then discharged. The subsequent day, patients were contacted by telephone to complete a 24-h post-experiment survey of minor/major complications and patient satisfaction.

### Data collection

#### Baseline data

Age, weight, BMI, premorbid conditions, structured survey, and smCPET measurements were prospectively collected during the preoperative evaluation visit. DASI-estimated peak METs and peak VO_2_ were calculated using recommended formulas. Brief smCPET measures of peak attained and extrapolated VO_2_, METs, GXCAP, GXCAP-VO_2_ slope, GXCAP exercise and recovery slopes, change in pulmonary perfusion (ΔETCO_2_), and ventilatory efficiency (VE/VCO_2_) were recorded.

#### Primary endpoint

The primary endpoint was EPCs, as measured by any recorded POMS event on postoperative day 1, 3, or 5 (POMS ≥1). POMS is an 18-item survey that measures minor and major EPCs across nine domains (pulmonary, infectious, renal, gastrointestinal, cardiovascular, neurological, haematological, wound, and pain).^2^^,^^34^ These measures were extracted from the electronic medical record by a trained study team member, with one point allocated per identified EPC. If participants were discharged before postoperative day 5, clinic notes were examined to tabulate any identified complications.

#### Secondary endpoints

Secondary endpoints data were collected from the electronic medical record up to postoperative day 30. Secondary endpoints included a sub-composite of the POMS-CPR, length of stay (h), and a 30-day composite of major postoperative complications (MPOCs) (physician-diagnosed pneumonia, surgical site infection, stroke, myocardial infarction, atrial fibrillation, and mortality).

### Postoperative Morbidity Survey score threshold selection

The threshold of POMS ≥1 was selected based on prior publications as a reliable measure for identifying postoperative morbidity, increased hospital bed occupancy, and prolonged length of stay (Fig 1).^2^

**Figure 1:**
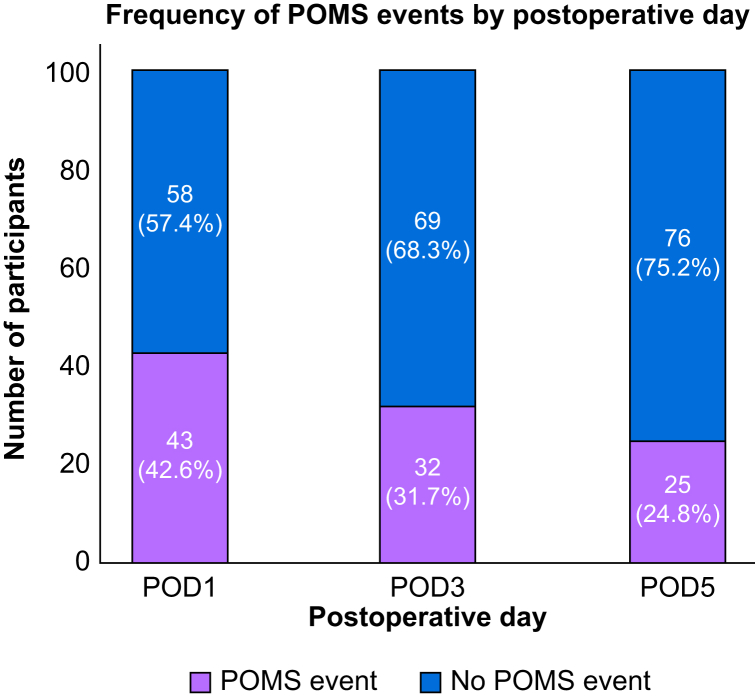
Postoperative Morbidity Survey (POMS) events as measured on postoperative day (POD) 1, 3, and 5.

### Statistical analysis

Descriptive statistics were calculated as follows: continuous variables are presented as mean and standard deviation (sd), or median and inter-quartile range (IQR), and categorical variables as frequency and percent. To identify relevant EPC predictors among structured survey measures, interrelated smCPET measures, and clinical variables, importance rankings were conducted using an elastic net regularisation machine learning model approach that integrates L1 and L2 penalties of the Lasso and Ridge methods.^35^ This technique reduces multicollinearity among the tested measurements, permitting the identification of relevant predictors of the primary and secondary endpoints. To prevent overfitting and improve model accuracy given the relatively small sample size, five-fold cross-validation was performed to select the optimal alpha and lambda values. Optimal model selection was assessed using root mean square error or area under the curve (AUC) of the receiver operating characteristic curve on a validation cohort of 20% of the original data set.

Important variables were identified and used in generalised linear models using negative binomial regression for count data (length of stay) and logistic regression models for binary data (POMS ≥1 and POMS-CPR ≥1). Based on machine learning model-identified feature importance, models were adjusted for *post hoc* Portsmouth Physiological and Operative Severity Score for the enUmeration of Mortality and morbidity (POSSUM) operative severity score for procedural risk and preadmission beta-blocker use.^36^ To reduce model overfitting, the number of covariates was restricted to a 1:10 ratio of covariates to outcome occurrences, with stepwise model selection used to refine the models. Effects of the EPC predictors were reported as adjusted odds ratios (ORs) with 95% confidence intervals (CIs). All statistical analyses were performed using R version 4.4.1 (R Foundation for Statistical Computing, Vienna, Austria). Statistical significance was set to *P*<0.05. Multiple comparison adjustment was performed using the Benjamini–Hochberg false discovery rate control procedure at a prespecified threshold of 5%.

#### Missing data

A complete-case analysis for missing data was used, where participants with missing data were excluded from analysis of the respective endpoint. Similarly, dropouts were removed from the analysis.

## Results

### Baseline characteristics

**Figure 2:**
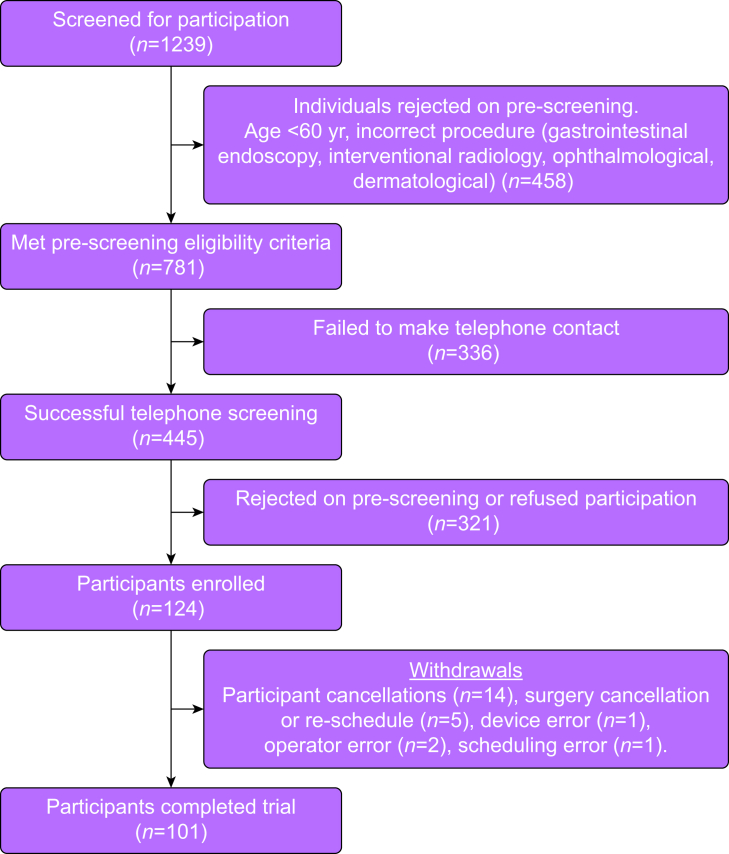
CONSORT participant study flow diagram.

During the study time period of June 2023 to June 2024, 1239 patients were screened and 124 participants were enrolled in the study (Fig 2). Twenty three participants (18.5%) of the enrolled cohort were excluded: participant cancellation (*n*=14) and surgery cancellation or rescheduling (*n*=9), for a final study cohort of 101 participants.

Of 101 participants, 53 (52.5%) had POMS ≥1 event. The median number of POMS events was 1 (IQR 0–2). Females comprised 43.6% and the median age was 69 yr (IQR 66–73), 6.9% reporting active smoking and 52.5% active alcohol use. The median revised cardiac risk index score was 1 (IQR 1–1), with 59/101 (58.4%) reporting essential hypertension, 6/101 (5.9%) atrial fibrillation, and 59/101 (58.4%) solid tumour on preadmission diagnoses (Table 1). Abdominal (64/101, 63.4%), thoracic (12/101, 11.9%), and other (18/101, 17.8%) procedures constituted the majority of procedures. Of 101 procedures, 71 (70.2%) were performed using minimally invasive approaches, with 99 (98%) performed under general endotracheal anaesthesia. The median operative time was 205 min (IQR 120–273) (Table 2). No intraoperative blood transfusion, cardiac complications, or deaths were reported. Median length of stay was 24.8 h (IQR 8–52.5). Participants who experienced postoperative morbidity (POMS score ≥1) had significantly longer hospital stays than those without complications (median 46 h [IQR 24–80] *vs* 20.5 h [IQR 6–26]; *P*<0.001).

**Table 1:** Baseline characteristics between participants with and without Postoperative Morbidity Survey. outcome ≥1.∗Unadjusted Student's *t*-test, or χ^2^ test as indicated. BMI: body mass index; Fisher's: Fisher's exact test; ACE/ARB, angiotensin converting enzyme and angiotensin receptor blocker; IQR, inter-quartile range; sd, standard deviation.

	No (48)		Yes (53)		Total (101)		Sig.∗	Fisher'sexact test
Variable								
Age (yr), median (IQR)	69	(66–73)	69	(66–73)	69	(66–73)	0.959	
Sex, *n* (%)								
Female	19	(39.6)	25	(47.2)	44	(43.6)		
Male	29	(60.4)	28	(52.8)	57	(56.4)	0.443	
BMI (kg m^−2^), mean (sd)	28.2	(6.6)	27.8	(6.4)	28.0	(6.4)	0.722	
Revised cardiac risk index,								
median (IQR)	1	(1–1)	1	(1–1)	1	(1–1)		
**Preoperative comorbidities, *n* (%)**								
Hypertension	27	(56.3)	32	(60.4)	59	(58.4)	0.674	
Hyperlipidaemia	30	(62.5)	26	(49.1)	56	(55.4)	0.175	
Pulmonary hypertension	1	(2.1)	0	(0)	1	(1)	0.291	0.475
Atrial fibrillation	0	(0)	6	(11.3)	6	(5.9)	**0.016**	
Heart failure, preserved ejection fraction	1	(2.1)	1	(1.9)	2	(2)	0.944	0.727
Heart failure, reduced ejection fraction	0	(0)	2	(3.8)	2	(2)	0.174	0.273
Myocardial infarction	1	(2.1)	4	(7.5)	5	(5)	0.206	
Cerebrovascular disease	0	(0)	2	(3.8)	2	(2)	0.174	0.273
Chronic obstructive pulmonary disease	6	(12.5)	4	(7.5)	10	(9.9)	0.405	
Asthma	6	(12.5)	8	(15.1)	14	(13.9)	0.706	
Obstructive sleep apnoea	7	(14.6)	8	(15.1)	15	(14.9)	0.943	
Chronic kidney disease	2	(4.2)	2	(3.8)	4	(4)	0.919	0.653
Electrolyte disorder	2	(4.2)	0	(0)	2	(2.0)	0.133	0.223
Diabetes mellitus	8	(16.7)	12	(22.6)	20	(19.8)	0.452	
Thyroid disease	5	(10.4)	9	(17)	14	(13.9)	0.340	
History of solid tumour	23	(47.9)	36	(67.9)	59	(58.4)	**0.042**	
Anaemia <8.8 g dl^−1^	0	(0)	1	(1.9)	1	(1)	0.339	0.525
**Social history, *n* (%)**								
Smoking history								
Former	21	(43.8)	18	(34)	39	(38.6)		
Active	2	(4.2)	5	(9.4)	7	(6.9)	0.422	
Marijuana								
Active	3	(6.3)	5	(9.4)	8	(7.9)	0.322	
Alcohol use								
Former	13	(27.1)	17	(32.1)	30	(29.7)		
Active	23	(47.9)	30	(56.6)	53	(52.5)	0.2	
**Cardioselective medication use, *n* (%)**								
Beta-blocker	9	(18.8)	25	(47.2)	34	(33.7)	**0.003**	
Calcium channel blocker	14	(29.2)	16	(30.2)	30	(29.7)	0.911	
ACE/ARB	23	(47.9)	22	(41.5)	45	(44.6)	0.518	
Diuretic	11	(22.9)	10	(18.9)	21	(20.8)	0.617

**Table 2:** Comparison of perioperative characteristics between participants with and without Postoperative Morbidity Survey (POMS) outcome ≥1. ∗Student's *t*-test, χ^2^, or Mann–Whitney *U* test as indicated. ^†^Laparoscopic or minimally invasive procedures: abdominal major, 58/64 (90.6%); musculoskeletal, 0/5 (0%); neurological, 1/2 (50%); thoracic major, 10/12 (83.3%); other, 2/18 (11.1%), Bonferroni-calculated *P*-value for multiple comparisons *P*<0.00625. IQR, inter-quartile range; sd, standard deviation.

	No (*n*=48)		Yes (*n*=53)		Total (*n*=101)		Sig.∗
Variable	*n*	(%)	*n*	(%)	*n*	(%)	
**Procedure type**^†^							
Musculoskeletal major	4	(8.3)	1	(1.9)	5	(5.0)	
Abdominal major	32	(66.7)	32	(60.4)	64	(63.4)	
Neurological major	1	(2.1)	1	(1.9)	2	(2.0)	
Thoracic major	0	(0)	12	(22.6)	12	(11.9)	
Other	11	(22.9)	7	(13.2)	18	(17.8)	**0.006**
**Anaesthesia type**							
General, natural airway	1	(2.1)	1	(1.9)	2	(2)	
General endotracheal anaesthesia (GETA)	44	(91.7)	48	(90.6)	92	(91.1)	
Regional + GETA	3	(6.3)	4	(7.5)	7	(6.9)	0.966
**POSSUM composite scores**							
Predicted mortality (%)	6.6	(5.8)	10.5	(8.7)	8.2	(6.8)	**0.01**
Predicted morbidity (%)	31.5	(18.9)	42.9	(23.1)	36.7	(21)	**0.008**
Physiological score (at the time of surgery)	19.5	(4.4)	20.3	(4.7)	19.8	(4.3)	0.389
Operative severity score (after surgery)	9.9	(3.3)	12.1	(3.9)	11.1	(3.8)	**0.003**
Operative time (min)							
Mean (sd)	171.5	(100)	257.3	(150.7)	216.5	(135.6)	**0.001**
Median (IQR)	169.5	(83.3–247.5)	237	(143.5–325)	205	(118.5–274)	
Length of stay (h)							
Median (IQR)	20.5	(6–26)	46	(24–80)	24.8	(8–52.5)	**<0.001**

### Study instrument findings

**Figure 3:**
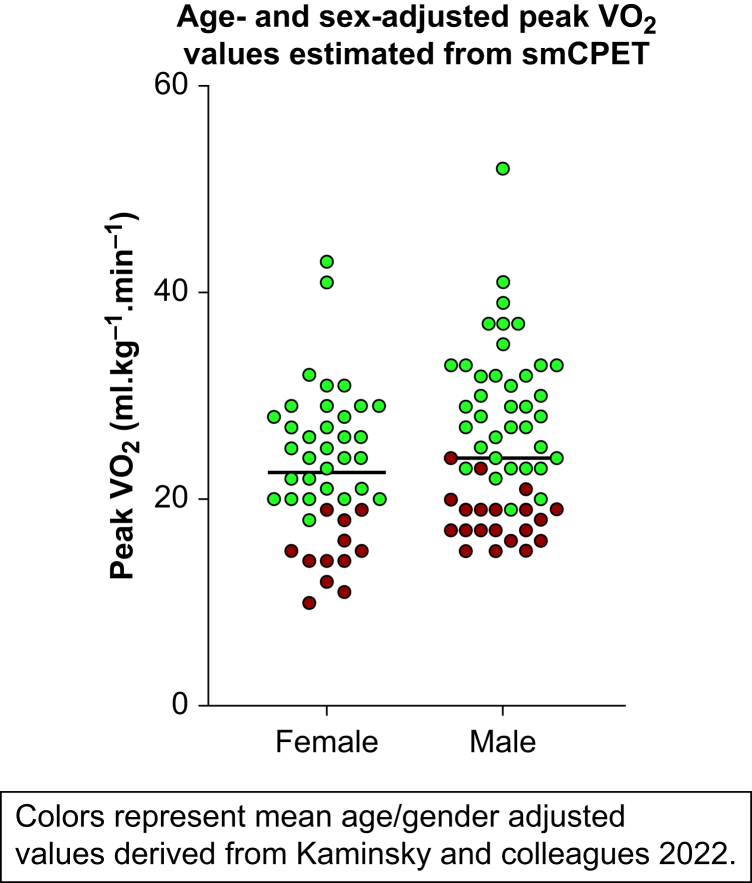
Age- and sex-adjusted submaximal cardiopulmonary exercise (smCPET)-derived peak VO_2_ in the studied cohort.

All participants endorsed the ability to reliably climb two flights of stairs (METs >4) during screening. The mean session time was 16.4 min (sd 5.3). During smCPET, peak respiratory exchange ratio (RER) was a mean of 0.9 (sd 0.1), indicative of submaximal effort (RER <1.05), and anaerobic threshold was met in 49/101 (48.5%) participants. The median subjective METs survey score was 8 METs (IQR 6–10), DASI-estimated METs was 8.9 (IQR 8.3–9.9). The median brief smCPET-derived extrapolated peak METs was lower at 6.8 (IQR 5.4–8.2). Peak GXCAP impairment (<3000 ml O_2_ kPa^−1^ beat^-1^) was observed in 17/101 participants. The overall mean smCPET-derived peak VO_2_ was 24.5 ml kg^−1^ min^−1^ (sd 7.6). When evaluating smCPET-derived peak VO_2_ measurements to age- and sex-adjusted ranges, 31.6% (*n*=32) of participants were below normal range values (Fig 3).^37^ No DASI-estimated VO_2_ values were below normal age- and sex-adjusted VO_2_ in the study cohort. Unadjusted GXCAP-VO_2_ slope in participants with and without POMS ≥1 was the only studied variable of significance (3.7 *vs* 4.2 min 100 beat^−1^^kPa^-1^,^ *P*=0.047; Table 3).

**Table 3:** Study measurements between participants with and without Postoperative Morbidity Survey outcome ≥1. ∗Student's *t*-test, χ^2^ test unless otherwise specified. ^†^Session time includes structured surveys + 6-min submaximal cardiopulmonary exercise test. ^‡^Independent samples Mann–Whitney *U* test. CPET, cardiopulmonary exercise test; DASI, Duke Activity Status Inde/x; HR, heart rate; IQR, inter-quartile range; METs, metabolic equivalents; sd, standard deviation; VE, minute ventilation; VO_2_, peak oxygen uptake; VCO_2_, carbon dioxide production.

Measurement	No (*n*=48)		Yes (*n*=53)		Total (*n*=101)		Sig ∗
**Subjective METs** (ml O_2_kg^−1^ min^−1^)							
Median (IQR)	8	(6–10)	8	(6–10)	8	(6–10)	NS
**Duke Activity Status Index**							
DASI sum	50.2	(9.0)	49.1	(9.5)	49.9	(9.2)	0.556
DASI-estimated VO_2_ (ml O_2_ kg^−1^ min^−1^), mean (sd)	31.2	(3.9)	30.7	(4.1)	31.1	(4.0)	0.557
DASI-estimated METs, mean (sd)	9.0	(1.1)	8.8	(1.2)	8.9	(1.1)	0.554
**Submaximal CPET**							
Session time,^†^ mean (sd)	16.9	(6.6)	16.0	(3.9)	16.4	(5.3)	0.423
Borg Survey of Perceived Exertion, on exercise testing^‡^							
Median (IQR)	2	(2–2.5)	2	(1–3)	2	(2–3)	0.234
Anaerobic threshold met during 3 min of exercise							
Yes, *n* (%)	20	(41.7)	29	(54.7)	49	(48.5)	0.19
**Submaximal CPET measures**							
Extrapolated peak VO_2_, mean (sd)	24.1	(7.1)	24.8	(8.1)	24.5	(7.7)	0.632
Extrapolated peak METs, mean (sd)	6.9	(2.0)	7.1	(2.3)	7.0	(2.2)	0.653
Ventilatory efficiency slope (VE VCO_2_^−1^)	28.2	(3.8)	27.6	(5.0)	28.0	(4.3)	0.571
Delta pulmonary perfusion (ΔETCO_2_, kPa)	0.6	(0.5)	0.5	(0.5)	0.6	(0.5)	0.315
Peak GXCAP (ml O_2_ kPa^−1^ beat^−1^)	4251	(1113)	4055	(1688)	4160	(1394)	0.499
GXCAP-VO_2_ slope (during exercise, min 100 kPa^-1^ beat^−1^)	4.2	(1.5)	3.7	(1.2)	3.9	(1.4)	**0.047**
GXCAP-time slope (during exercise, ml O_2_ min kPa^-1^ beat^−^^^2^^)	18.4	(6.7)	16.2	(8.8)	17.3	(7.8)	0.161
GXCAP recovery slope (during recovery, ml O_2_ min kPa^-1^ beat^−^^^2^^)	4.9	(8.1)	4.4	(11.8)	4.9	(9.8)	0.793

### Machine learning analysis

An elastic net regularisation approach characterised predictors of the primary endpoint using root mean square error or AUC criteria was used for continuous or binary outcomes, respectively, to select an optimal regression model across 32 clinically relevant features (Supplementary Fig. S1). Training results determined Lasso regression as the optimal model, with moderate performance on the test set (accuracy 0.67, precision 0.6–0.73). Hyperparameter optimisation was performed using five-fold cross-validation, yielding optimal parameters of α=0.18 and L1 ratio of 0.1. Feature importance was analysed using standardised beta coefficients, identifying clinically relevant predictors of POMS ≥1 (top three in order of importance: beta-blocker use, GXCAP-VO_2_ slope, POSSUM operative severity). Remaining variables were eliminated through regularisation (coefficient 0) suggesting minimal predictive value for POMS ≥1. Multivariable logistic regression models were then guided by the feature importance rankings using stepwise model selection.

### Primary endpoint

Of 101 participants, 53 (52.4%) had POMS ≥1 event. After adjustment for POSSUM operative severity and beta-blocker use, GXCAP-VO_2_ slope was associated with POMS ≥1 (OR_adj_ 0.94; 95% CI 0.89–0.99; *P*=0.011). For each unit decrease in GXCAP-VO_2_ slope, the odds of POMS ≥1 increased by 6.4%. Hosmer–Lemeshow goodness of fit was indicative of good model fit (*P*=0.16) with a McFadden's pseudo R^2^ of 0.18 (moderate fit). A receiver operating characteristic demonstrated that, with these three measures, the model showed good discrimination (AUC of 0.79), with a sensitivity of 0.93 and specificity of 0.63. POSSUM operative severity was the strongest predictor (0.79), followed by beta-blocker use (0.7) and GXCAP-VO_2_ (0.68). The AUC remained consistent and reliable after bootstrapping (1000 iterations; mean AUC 0.79; 95% CI 0.69–0.88). The optimal threshold values for POMS ≥1 in our study cohort were as follows: POSSUM operative severity >9 (risk ratio 2.15), GXCAP-VO_2_ <25.2 (risk ratio 1.59), and beta-blocker use (yes).

### Secondary endpoints

Of 101 participants, 33 (32.7%) had POMS-CPR ≥1 events. After adjustment for POSSUM operative severity and beta-blocker use, GXCAP-VO_2_ slope was associated with POMS-CPR (OR_adj_ 0.931; 95% CI 0.88–0.99; *P*=0.015). Hosmer–Lemeshow (*P*=0.78) and McFadden's Pseudo R^2^ (0.178) indicated good model fit. For each unit decrease in GXCAP-VO_2_ slope, the odds of POMS-CPR ≥1 increased by 7%. Receiver operating characteristic analysis demonstrated an AUC of 0.78, with a sensitivity of 0.67 and specificity of 0.824. Bootstrapping demonstrated similar consistency and reliability (1000 iterations; mean AUC 0.79; 95% CI 0.68–0.88). After adjustment for POSSUM operative severity and procedural category, decreasing GXCAP-VO_2_ slope (OR_adj_ 0.98; 95% CI 0.96–0.99; *P*=0.01) was associated with prolonged length of stay.

### Exploratory endpoints

Lower smCPET-derived peak extrapolated VO_2_ (*P*=0.029) and METs (*P*=0.031) were associated with the presence of 30-day composite MPOCs (composite surgical site infection, pneumonia, myocardial infarction, stroke, major cardiac dysrhythmia), but subjective METs (*P*=0.218), DASI sum (*P*=0.609), DASI METs (*P*=0.705), DASI VO_2_ (*P*=0.608) were not significantly associated.

Treatment effects were evaluated across one primary and two secondary endpoints using the Benjamini–Hochberg procedure to control the false discovery rate. After correction, the primary endpoint (*P*=0.011) and both secondary endpoints (*P*=0.015 and *P*=0.01, respectively) remained statistically significant at a false discovery threshold of 5% (all adjusted q=0.015).

## Discussion

This study characterises brief smCPET measures for the identification of increased EPC risk in a low-morbidity surgical population. Machine learning methods identified GXCAP-VO_2_ slope as the optimal measure for detecting increased EPC risk, as measured by POMS ≥1.

The brief smCPET identified 17 participants (16.8%) with mild GXCAP impairment (peak GXCAP <3000 ml O_2_ kPa^−1^ beat^−1^) and nine participants (8.9%) with abnormal GXCAP (<2250 ml O_2_ kPa^−1^ beat^−1^ ). GXCAP correlates with invasive pulmonary capacitance measurement (r=0.86), a prognostic factor for pulmonary hypertension mortality, and outperforms echocardiography in the discrimination of pulmonary hypertension phenotypes.^21^^,^^22^^,^^38^ Impaired GXCAP is a strong predictor for pulmonary hypertension in patients with interstitial lung disease (AUC 0.84, *P*<0.001).^21^ Pulmonary hypertension has a well characterised deleterious impact on perioperative outcomes, and these findings suggest subclinical pulmonary capacitance impairment is frequently present before surgery.^39^

In our relatively healthy cohort, peak VO_2_ was not identified as an important feature for POMS ≥1, but a lower GXCAP-VO_2_ slope was. This slope encompasses several characteristics important for surgical fitness (Supplementary Fig. S2). A steeper slope likely represents superior pulmonary vascular recruitment and distensibility during dynamic exercise, suggesting a healthy pulmonary vasculature with significant reserve capacity and appropriate pulmonary vascular recruitment relative to metabolic demands. If the pulmonary system cannot increase the capacity proportionally to oxygen demands, because of pulmonary vascular disease, right ventricular dysfunction, or reduced pulmonary capillary density (pulmonary fibrosis, restrictive lung disease), particularly when the cardiopulmonary system is stressed and needs to accommodate increased blood flow, there may be an increased vulnerability to perioperative haemodynamic perturbations, such as the ability to handle perioperative fluid shifts. Thus, this novel marker may reflect effective optimisation of both right and left ventricular function, where lower values increase the likelihood of vulnerability to EPCs tabulated by the POMS-CPR (hypertension, respiratory insufficiency requiring oxygen therapy, oliguria). Furthermore, published reports have shown that GXCAP demonstrates modest correlation to diffusion capacity for carbon monoxide and peak VO_2_, implying that several steps for optimal ventilation–perfusion matching are encapsulated by the GXCAP-VO_2_ slope, including appropriate recruitment of pulmonary blood flow during stress, adequate gas exchange (as measured by appropriate increase in end-tidal CO_2_ during exercise [ΔETCO_2_]) and adequate stroke volume (as reflected by O_2_ pulse).^40^ The GXCAP-VO_2_ slope captures dynamic vascular function, potentially better revealing vulnerabilities to perioperative haemodynamic stress that are missed by static peak CPET measurements.

Furthermore, pulmonary capacitance is an important measure of cardiopulmonary health. Both invasive and echocardiographic-derived pulmonary capacitance are valuable predictors of all-cause mortality in pre- and postcapillary pulmonary hypertension and chronic heart failure, notably in patients with preserved ejection fraction.^41^^,^^42^ Among patients with chronic heart failure, lower pulmonary capacitance serves as an important marker for decompensation and rehospitalisation risk.^43^^,^^44^ Respiratory insufficiency or failure is the most common perioperative adverse event in patients with pulmonary hypertension, and GXCAP-VO_2_ may be capturing dynamic subclinical pulmonary capacitance impairment.^45^ This is notable, as the most common early postoperative pulmonary complication is persistent supplemental oxygen requirement (19.6%), and even a single postoperative pulmonary complication has been associated with increased early postoperative mortality, ICU admission, and extended length of stay.^46^

With self-reported functional capacity of >4 METs and revised cardiac risk index score ≤2, the study participants would not have likely received extensive preoperative evaluation. The peak values from structured surveys (subjective METs, DASI-estimated METs) and submaximal-derived peak extrapolated METs did not discriminate participants with or without POMS ≥1. However, smCPET identified more participants (11/101, 10.9%) failing to achieve 4.6 METs, when compared with subjective METs (6/101, 5.9%) and DASI surveys (0/101, 0%). In exploratory findings, smCPET-derived peak VO_2_ (*P*=0.029) and METs (*P*=0.031) outperformed subjective METs (*P*=0.218), DASI sum (*P*=0.609), DASI METs (*P*=0.705), and DASI VO_2_ (*P*=0.608), in the identification of a composite of MPOCs (surgical site infection, pneumonia, myocardial infarction, stroke, major dysrhythmia). These preliminary findings suggest enhanced discrimination of major perioperative complications and warrant further investigation.

This study found that GXCAP-VO_2_ slope better discriminated EPC risk in study participants, when compared with structured surveys and smCPET peak estimated values of METs and VO_2_. Compared with peak values, slope measures are protocol-independent and may better reflect the cardiopulmonary system's adaptability to the submaximal stress of surgery (e.g. blood loss, third space fluid shifts, hypotension) and postoperative recovery (e.g. fluid overload, active fluid removal, inadequate tissue perfusion). Prior published reports support this rationale and CPET slope measures have shown superior risk prediction for various cardiopulmonary conditions.^47^^,^^48^

These findings suggest that augmenting usual preoperative screening processes with precise cardiopulmonary performance data may be useful to identify preoperative patients at higher EPC risk. Brief smCPET is a cost-effective method that could help identify surgical patients requiring additional resource allocation, potentially leading to significant cost savings given that even a single postoperative complication substantially increases hospital cost.^49^ Although requiring further validation, a quantitative smCPET-driven approach could facilitate research methods for the development of automated clinical decision support systems that enhance early EPC risk detection and mitigation.

This study has limitations, including a small sample size (∼10% of eligible patients were enrolled), and a single-centre design, limiting its generalisability. Open-label trials may introduce biases given that both participants and researchers are aware of treatment allocation, although care providers were not. To minimise measurement bias, measurements were performed at the same site, with device calibration performed at the manufacturer's recommended schedule. Selection bias may be introduced with the stringent revised cardiac risk index inclusion criterion (≤2) which generated a relatively healthy cohort. Between-subject variability was minimised by model adjustment for operative and physiological differences in the study cohort, but risk for unknown confounders is present. Elastic net regularisation, although useful for handling multicollinearity, may be less stable for small datasets as cross-validation has fewer samples to establish reliable penalty parameters. Although the use of false discovery rate control demonstrated that all selected endpoints were significant, it inherently accepts a proportion of false positive results, which may cast doubt in clinical decision-making contexts. Of note, the analysis was not sufficiently powered to assess MPOC, and the reported findings should be considered exploratory. These limitations emphasise the need to cautiously interpret our findings and require robust clinical trials for external validation.

## Conclusions

Implementation of preoperative smCPET may enhance preoperative EPC risk screening, especially in patients with lower morbidity. Although requiring further validation, the GXCAP-VO_2_ slope was identified as a compelling investigational target for EPC risk. Brief smCPET excels through swift assessment (6 min), and comprehensive data acquisition. As an alternative to structured surveys, or possibly CPET, these advantages should be considered when evaluating the merit of a smCPET-driven approach to preoperative EPC screening.

## Authors’ contributions

Conceptualisation: ZJC, PH.

Investigation: ZJC.

Data curation: ZJC, JC, ABB, AJ, EG,

Funding acquisition: ZJC, PH.

Methodology: ZJC, HH, HML, PH.

Formal analysis: HH, HML.

Software: HH, HML.

Statistical analysis: HH, HML.

Data interpretation: JZ, FG, WST.

Writing – original draft: ZJC, JC, PH.

Writing – review and editing: JC, ABB, JZ, FG, WST.

## Funding

Shape Medical Systems, Inc., MN, USA (Grant#23-003942).

## Data availability statement

The data that support the findings of this study contain sensitive patient information that could compromise research participant privacy. A deidentified dataset is available from the corresponding author upon reasonable request, subject to institutional review board approval and verification of appropriate data use agreements. Requests will be reviewed on an individual basis to ensure compliance with patient privacy protections and institutional policies.

## Declarations of interest

ZJC declares partial research funding from Shape Medical Systems, Inc (MN, USA). PMH declares consulting for Cardiage LLC and Baudax Bio, consulting and sponsored research for Edwards Lifesciences, and equity interest in emka Medical.

## Figures and Tables

**figs1:**
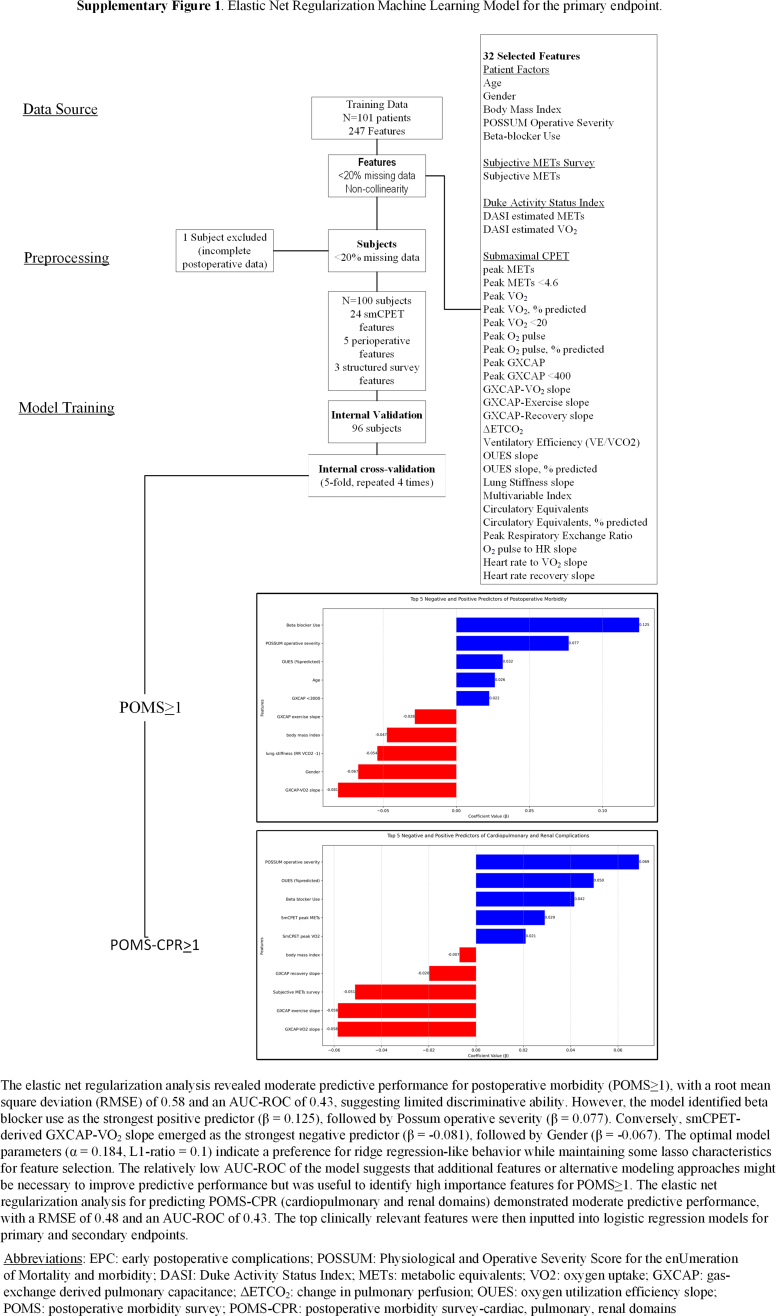


**figs2:**